# Pre-adult attention-deficit/hyperactive disorder and later adverse health outcomes: the 1987 Finnish birth cohort study

**DOI:** 10.1038/s41380-026-03700-6

**Published:** 2026-07-13

**Authors:** G. David Batty, Mika Gissler, Seyed Ehsan Mousavi, Varun Warrier, Tamsin Ford, Markus Keski-Säntti

**Affiliations:** 1 Department of Epidemiology and Public Health, University College London, London, UK; 2 Department of Data and Analytics, Finnish Institute for Health and Welfare, Helsinki, Finland; 3 Academic Primary Health Care Centre, Region Stockholm, Stockholm, Sweden; 4 Department of Molecular Medicine and Surgery, Karolinska Institutet, Stockholm, Sweden; 5 Neurosciences Research Center, Tabriz University of Medical Sciences, Tabriz, Iran; 6 Social Determinants of Health Research Center, Tabriz University of Medical Sciences, Tabriz, Iran; 7 Department of Psychiatry, University of Cambridge, Cambridge, UK; 8 Department of Psychology, University of Cambridge, Cambridge, UK

**Keywords:** ADHD, Depression

## Abstract

Whereas attention-deficit/hyperactive disorder (ADHD) has consistently been shown to be correlated with later risk of depression, anxiety, and substance misuse, the relationship with other adverse health endpoints, particularly somatic conditions, is uncertain. In a full-nation birth cohort study, we used a phenotype-wide approach to explore the influence, if any, of a ADHD diagnosis in childhood/adolescence on later disease and injury. Comprising 52594 children (25463 females) born in a single year with non-missing data, the 1987 Finnish Birth Cohort was generated from linkage of routinely collected data. ADHD diagnosis was captured from in- and out-patient hospital records up to age 18 years and study members continued to be surveilled for other diagnoses until 2020 (aged 33). Pre-adulthood, 0.43% (*N* = 228) of study members were diagnosed with ADHD. In people with ADHD, relative to population controls, there was a heightened risk of developing all of the 17 health endpoints examined. Of these associations, however, eleven were statistically significant after adjustment for age and sex, and five after further control for birth weight, family socioeconomic circumstances, study members’ educational performance, and multiple testing (odds ratio; 99.7%): neurotic disorders (2.11; 1.25, 3.41); substance abuse disorder (2.26; 1.27, 3.79); poisoning (2.30; 1.02, 4.52); mood disorders (2.45; 1.49, 3.88); and epilepsy (4.55; 1.82, 9.55). Repeating the main analyses following data imputation (*N* = 54045) confirmed these associations with the emergence of a connection between psychotic disorders and antecedent ADHD (2.63; 1.34, 5.18). Overall, young people with ADHD in the present cohort experienced an increased future burden of psychiatric and neurological conditions but seemingly not somatic disorders.

## Introduction

First posited over four decades ago, the developmental origins of adult disease – the notion that adverse health in later life is influenced by childhood exposures – has traditionally focused on the deterministic role of markers of physical development [1], such as body weight from birth to adolescence, physical stature, and age at onset of puberty [2–5]. By contrast, it is only in the last ten years that the role of neurological development and associated disorders such as intellectual disability, autism, Tourette syndrome/Tic disorder, and attention-deficit/hyperactive disorder (ADHD) in the occurrence of adult disease and injury have been systematically examined [6–9]. Of these, if shown to be connected to later health, ADHD holds the largest potential for event prevention given that it is the most commonly occurring neurodevelopmental disorder (3–7% prevalence [10]) and, indeed, the most prevalent childhood psychiatric condition overall; seemingly increasing in occurrence [11]; and its core symptoms of age-inappropriate levels of distraction, restlessness, and impulsivity are modifiable via prescription psychostimulants and psychosocial intervention [12].

There is a clear *prima facie* case for ADHD increasing the future risk of an array of adverse health events. First, the prevalence of unfavourable lifestyle factors – obesity, cigarette smoking, heavy alcohol intake – plus educational challenges and poverty is markedly greater in people with ADHD relative to their unaffected peers [13, 14]. Plausibly via these mechanistic pathways, people with this neurodevelopmental disorder appear to have a greater burden of mental illness and addiction problems such as depression [15, 16], anxiety [17], and substance misuse [18]. Whether other conditions which are known to be patterned by lifestyle characteristics [19] and socioeconomic circumstances [20] are also more common in people with a diagnosis of ADHD requires clarification.

Second, perhaps owing to the core symptoms of ADHD, there are also reasons to anticipate associations with common bacterial and viral infections, unintentional injury, and neurological conditions. Thus, perhaps through a lack of compliance with preventative measures such as mask wearing and handwashing, ADHD-affected individuals have been shown to have a higher burden of COVID-19 compared with the general population [21]; the connection with other common pulmonary infections, such as influenza and pneumonia, is largely untested however. Unintentional injury, such as road traffic accidents [22] may also be more common in people with this neurodevelopmental disorder because of inattention, impulsivity, and longer reaction time [23], although the impact, if any, of this neurodevelopmental disorder on other commonly occurring accidents such as poisonings is poorly understood. Lastly, to date, while  neurological conditions such as epilepsy would seem to be associated with the development of ADHD – aggregated estimates across studies suggest a quadrupling in risk [24] – the converse, that is the role of antecedent of ADHD, is largely untested.

To address the paucity of evidence for ADHD as a potential risk factor for these health outcomes, we undertook analyses of a full nation birth cohort of individuals born in Finland in 1987 using routinely collected data on ADHD, covariates, and hospital-recorded episodes of adverse health. The present dataset also allow us to examine the potential explanatory role of confounding factors, such as socioeconomic disadvantage, and the mediating influence, if any, of educational performance – characteristics which are both a consequence of ADHD [25] and risk factors for a series of health conditions [20].

## Methods

**Fig. 1:**
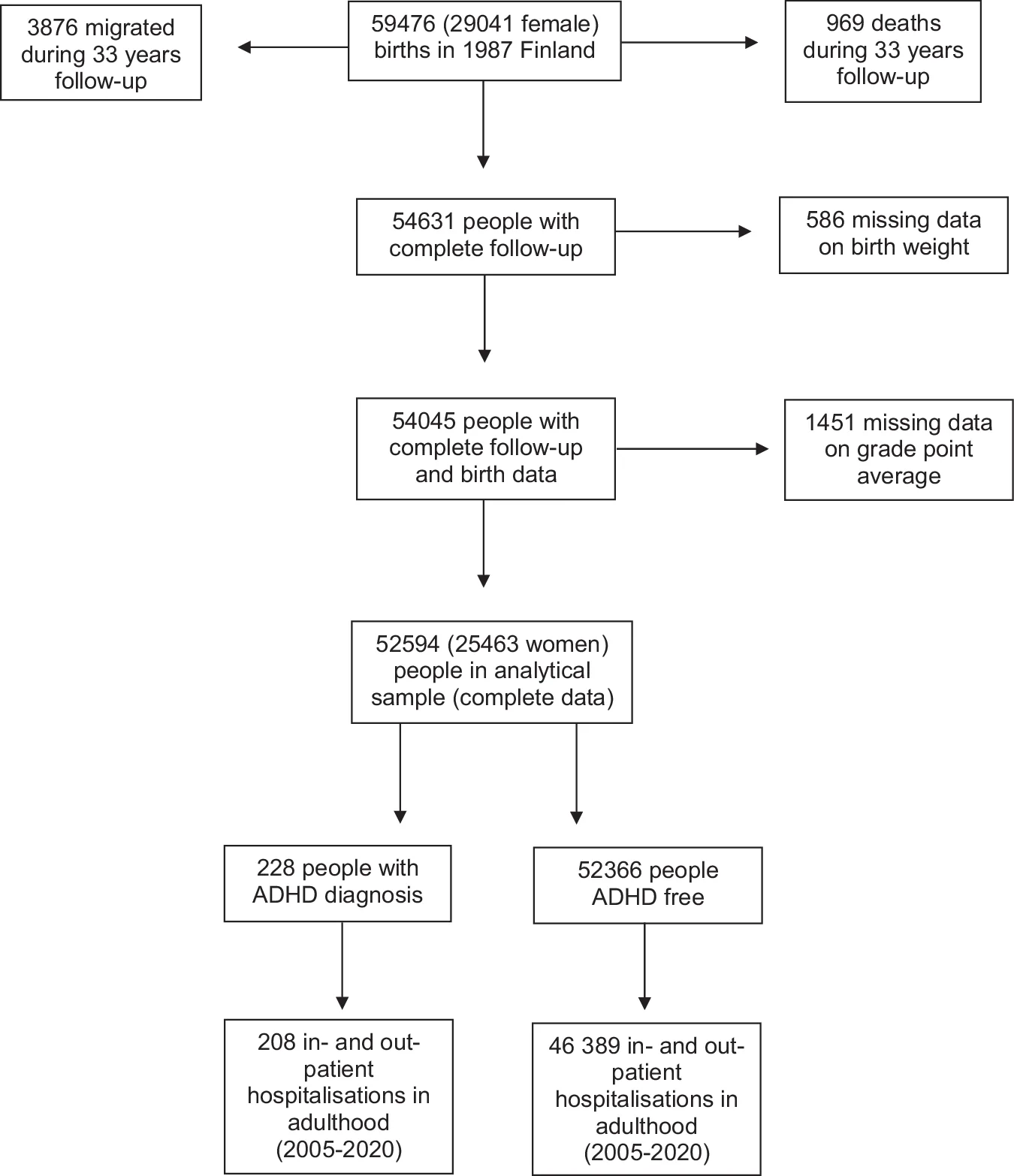
Flow of study members into the analytical sample. Beginning with all births occuring in Finland in 1987, for the present cohort, the figure displays number of people lost to follow-up via migration and death plus those with missing data.  It also depicts the number of hospital visits made during follow-up in people with and without ADHD.

Described in detail elsewhere [26], the 1987 Finnish Birth Cohort was generated from the linkage of routinely collected data using the personal identification number allocated to every citizen. This was the year in which the Finnish Medical Birth Registry was first formed, so providing important data on the study members and their families and, simultaneously, individual-participant data on hospitalisations for somatic outcomes became available. The cohort comprises 59476 Finland-born individuals (Fig. 1). Study approval was provided by the Ethical committee at the Finnish Institute for Health and Welfare (§28/2009). The composition of this manuscript conforms to the Strengthening the Reporting of Observational Studies in Epidemiology (STROBE) Statement [27].

### Ascertainment of ADHD and health outcomes

**Fig. 2:**
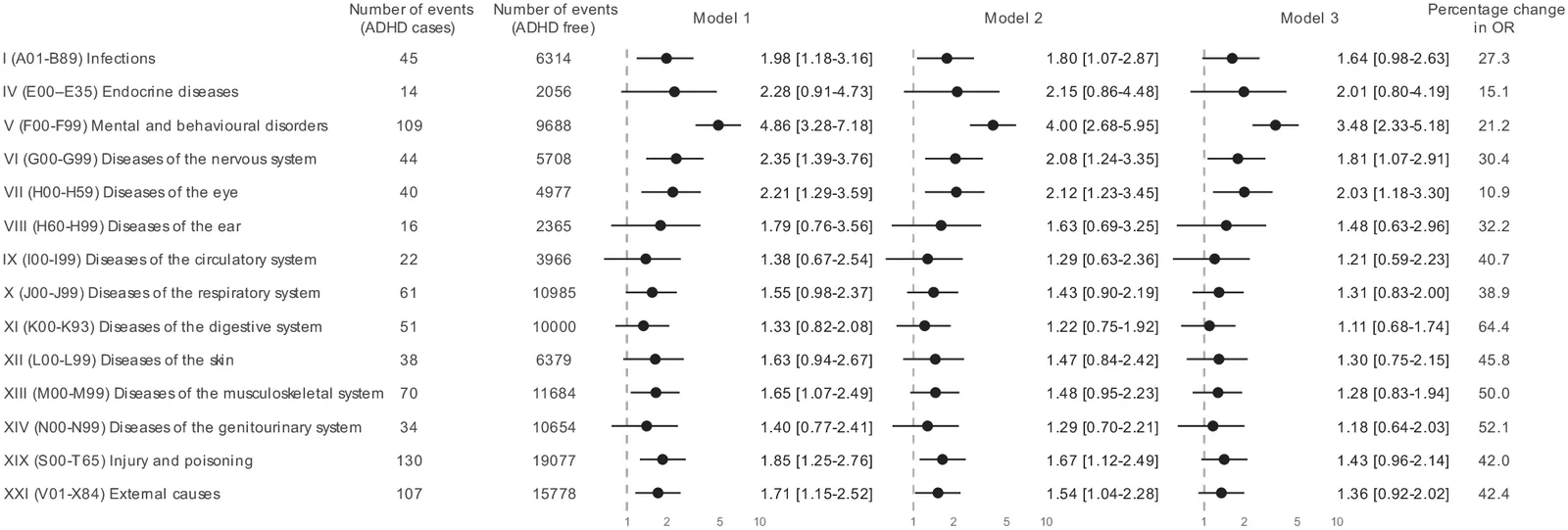
Odds ratios (99.6% confidence intervals) for the association between ADHD diagnosis and subsequent hospitalisation for 14 health conditions (N = 52594). Odds ratios (x-axis) are the odds of a given outcome in people with an ADHD diagnosis versus the those without (the referent). Model 1 comprises sex; Model 2 sex, family socioeconomic status, and birth weight; and Model 3 all described variables plus educational performance. As a birth cohort, there is no variation in age in the study sample, so no such adjustment was required. All effect estimates are corrected for multiple testing. Percent attenuation is a comparison of effect estimates in Model 3 and Model 1.

**Fig. 3:**
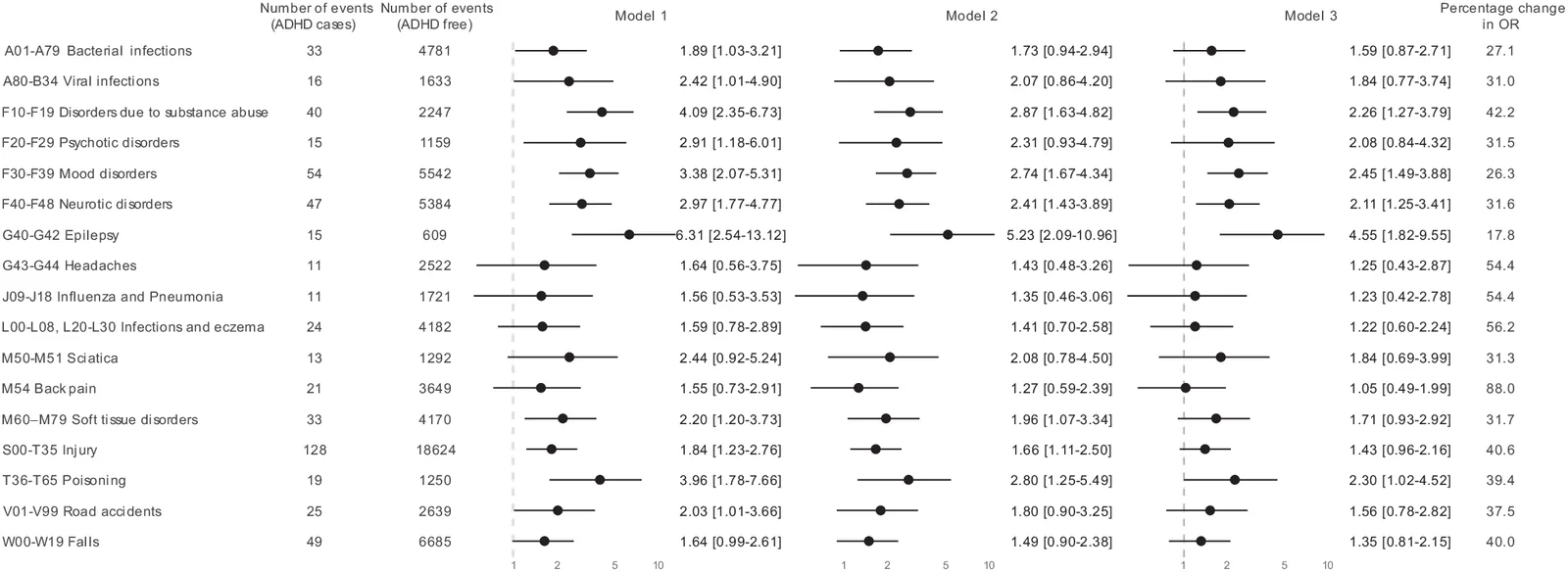
Odds ratios (99.7% confidence intervals) for the association between ADHD diagnosis and subsequent hospitalisation for 17 health conditions (*N* = 52594). This figure depicts a disagregation of the outcomes in Fig. 2. Odds ratios (x-axis) are the odds of a given outcome in people with an ADHD diagnosis versus the those without (the referent). Model 1 comprises sex; Model 2 sex, family socioeconomic status, and birth weight; and Model 3 all described variables plus educational performance. As a birth cohort, there is no variation in age in the study sample, so no such adjustment was required. All effect estimates are corrected for multiple testing. Percent attenuation is a comparison of effect estimates in Model 3 and Model 1.

The Finnish Care Register for Health Care (formerly the Hospital Discharge Register) captures in- (1969-present) and out-patient care episodes (1998-present) from all hospitals in the country [28]. Coded according to the International Classification of Diseases (ICD) versions 9 (1987–1995) and 10 (1996–2004), a diagnosis of ADHD was denoted by codes 314 (ICD-9) and F90 (ICD-10). ICD codes for the health outcomes in the present analyses are given in Figs. 2 and 3.

### Assessment of covariates

Sex and birth weight were extracted from data entered into the Medical Birth Registry at birth in 1987. Parental socioeconomic status, located in the Register of Social Assistance for the period 1987 (birth) to 2004 (age 16–17), was denoted by the duration (months) the study members’ carer, typically the parent, was in receipt of government income support. Longer duration was used to denote greater socioeconomic disadvantage. For educational performance of the study member, we used school grade point average (score range: 4–10). Provided by the Finnish National Agency for Education, these data were taken from the study members’ application to attend secondary school – the application is required if secondary school attendance is sought which is typically the case. In practice, most applications were made in Spring 2003 (aged 15–16). A higher grade point average is indicative of higher educational performance.

### Derivation of the analytical sample, statistical analyses, and code availability

From the 59476 births (29041 females) in 1987, we excluded people who subsequently died or emigrated before the end of follow-up 33 years later (31 December 2020) and those missing covariate data (Fig. 1). Analyses were therefore based on a sample of 52594 individuals (25463 women). In this group, a ADHD diagnosis on or before their 18th birthday (1987–2005) was related to the occurrence of adverse health events thereafter with censoring at age 33 (2005–2020). During follow-up, a study member could conceivably not have diagnosis of a health episode, a single diagnosis, or multiple diagnoses made on single or multiple occasions. When there were multiple events recorded, each contributed separately to the analyses of a specific health outcome. Thus, in preliminary analyses, of the 228 individuals with ADHD diagnosis, during 15 years of follow-up, 208 had one or more contact with hospital services, with the corresponding count in the ADHD-free group of 52366 being 46389.

We used logistic regression analyses to computed odds ratios to summarise the relationship between ADHD and later event diagnosis. Taking a phenotype-wide approach, those outcomes with 10 or more events in the ADHD group and population controls qualified for inclusion in our analyses. In minimally-adjusted analyses (model 1), we controlled odds ratios for sex; with this being a single-year birth cohort, no adjustment for age was required. Further adjustment was then made for the family socioeconomic status and birth weight (model 2). Lastly, to model 3, we added educational performance, a potential mediating factor.  To take into account multiple testing, a Bonferroni correction was applied to the computation of all effect estimates, with statistical significance set at p = 0.004 (96.6% confidence intervals) for the analyses featured in Fig. 2 on ICD chapters (14 outcomes) and *p* = 0.003 (99.7 confidence intervals) for the analyses featured in Fig. 3 for chapter disaggregation (17 outcomes) [29]. Change in odds ratios following adjustment for multiple covariates relative to minimal correction was computed using standard formulae ([ln odds ratio[Model 1] – ln odds ratio [Model 3]] / ln odds ratio[Model 1]) with the product expressed as a percentage [30]. Computer syntax is available from the authors (MK-S).

## Results

In the analytical sample of 52594 individuals, 0.4% (*N* = 228) were diagnosed with ADHD on or before their 18^th^ birthday (Table 1). Relative to their unaffected counterparts (51%), study members with ADHD were more likely to be male (86%). Individuals with ADHD were typically disadvantaged relative to the general population, such that they were marginally smaller at birth as indexed by birth weight; more likely to have been in a family in receipt of financial aid, as well as requiring a longer duration of support; and have an increased likelihood of having a lower grade point average.

**Table 1:** Study member characteristics according to ADHD diagnosis: The 1987 Finnish Birth Cohort (*N* = 52594).

	ADHD	
	Yes	No
Population at risk, N (%)	228 (0.4)	52366 (99.6)
Male, N (%)	197 (86.4)	26934 (51.4)
Parents receiving any social income, N (%)	139 (61.0)	19145 (36.6)
Duration in receipt of social income, months (mean, SD)	47.3 (73.4)	17.3 (44.6)
Grade point average (mean, SD)	6.9 (0.8)	7.8 (0.9)
Birth weight, g (mean, SD)	3462.1 (772.7)	3571.9 (546.9)

In Fig. 2, we depict the association between ADHD and subsequent hospitalisation for outcomes denoted by 14 ICD chapters. In analyses corrected for sex and multiple comparisons (model 1), there was a higher risk of all 14 endpoints in people diagnosed with ADHD relative to population controls, although statistical significance was only apparent for half. Where the confidence interval did not include unity, the magnitude of the odds ratio (99.6% confidence interval) ranged between 1.65 (1.07, 2.49) for diseases of the musculoskeletal system and 4.86 (3.28, 7.18) for mental and behavioural disorders. Adjustment for socioeconomic background and birth weight (model 2) and, separately, educational performance (model), produced a similar, stepwise, attenuation in these point estimates (range comparing Model 1 with Model 3: 11–50%). Only three ADHD–health associations remained statistically significant in Model 3 (ascendingly): diseases of the nervous system (1.81; 1.07, 2.91); diseases of the eye (2.03; 1.18, 3.30); and mental and behavioural disorders (3.48; 2.33, 5.18).

Next, in Fig. 3 we show the results of our primary analyses: the association of ADHD diagnosis with 17 outcomes disaggregated from the ICD chapters in Fig. 2. Again, a higher risk of all these endpoints was seen in people with ADHD, however, eleven of these associations were statistically significant (99.7% confidence interval) after minimal adjustment and Bonferroni correction (model 1). After taking into account differences in parental socioeconomic status, study members’ birth weight, and education achievement (Model 3), five associations remained (ascendingly): neurotic disorders (2.11; 1.25, 3.41); substance abuse disorder (2.26; 1.27, 3.79); poisoning (2.30; 1.02, 4.52); mood disorders (2.45; 1.49, 3.88); and epilepsy (4.55; 1.82, 9.55).

We carried out four sets of sensitivity analyses. First, we carried out some context-specific analyses. The low absolute number of hospital events in the small group of girls (*N* = 31) with an ADHD diagnosis prevented sex-stratification. We were, however, able to explore ADHD associations according to receipt of income support, our indicator of family socioeconomic circumstances. We computed *p*-values for interaction for sex-adjusted odds ratio for the 14 chapter outcomes (Fig. 2) and the disaggregation into 17 endpoints (Fig. 3) and, of these, effect modification was only apparent for Mental and behavioural disorders in relation to earlier ADHD (p-value for interaction 0.002). That is, while gradients were directionally consistent, in people in receipt of social income (*N* = 19284) there was a markedly stronger association with ADHD (odds ratio; 99.6% CI: 5.85; 4.16, 8.31) than in study members (*N* = 33310) who did not receive such financial aid (2.41; 1.47, 3.83).  This solitary interaction effect from the many examined is likely to be a chance result however.

Next, we explored if the ADHD associations were sensitive to period of follow-up. To do so, we created a partition at age 25.5 years (18–25.5; >25.5–33.0) then recomputed the main analyses. In doing so we focused on new events; thus, if a study member had an event in the first period of follow-up, they did not feature in analyses for that outcome in the second period of follow-up. Only those outcomes with 10 or more events in the exposed group during follow-up qualified for inclusion. All associations were directionally consistent with those in the main analyses although statistical significance was lost on occasion, perhaps owing to the lower power (Supplemental Fig. 1A, B, and Fig. 2A, B). Third, given that our cohort was generated via the linkage of routinely collected data, missing data was uncommon, and certainly not of the level apparent in comparable field-based studies. Nonetheless, for the key explanatory variable of educational performance based on grade point average data, 2.7% of people (*N* = 1451) were missing data (Fig. 1). Using sex, parental cohabitation, and family socioeconomic status, we therefore computed  missing data on grade point average using multiple imputation by chained equations with predictive mean matching (m = 20). From the original 52594 in the non-missing sample, this increased our sample size to 54045 and all statistical models were re-run using this sample; we also included parental cohabitation as a new covariate to further test the robustness of our results. Risk estimates obtained from the imputed data sets were pooled using well described criteria [31]. As expected, this slightly larger sample size produced somewhat more precise estimates as evidenced by the marginally tighter confidence intervals, but the pattern of results was essentially unchanged. There were slightly higher effects estimates for the three statistically significant associations (model 3) for the main event chapters (Supplemental Fig. 3A). In the disaggregation of these outcomes (Supplemental Fig. 3B), again, there was no change to the overall pattern of results with the exception of the emergence of psychotic disorders (2.63; 1.34, 5.18).

Fourth, to control for multiple testing of ADHD–health correlations, we used the Bonferroni correction which may be regarded as an overly conservative approach. In sensitivity analyses, we therefore also used the more liberal Benjamini-Hochberg procedure which identified an additional five statistically significant relationships (Supplemental Table 1): M60–M79 Soft tissue disorders; F20-F29 Psychotic disorders; S00-T35 Injury; A01-A79 Bacterial infections; and A80-B34 Viral infections.

Lastly, to increase confidence in our new results for hospitalisations and antecedent ADHD, we attempted to replicate well-explored predictors of mortality in the same population (Supplemental table 2). In sex-adjusted analyses, men (versus women odds ratio; 95% confidence interval: 2.95; 2.46, 3.56), study members with lower educational achievement (low versus high grade point average: 2.37; 2.01, 2.79), those from socioeconomically disadvantaged families (per SD longer duration of family income support: 1.28; 1.21, 1.34), and those diagnosed with ADHD (versus none: 2.15; 0.97, 4.08) all experienced a higher risk of premature mortality.

## Discussion

The main finding of the present study was that, after taking into account covariates and multiple testing in a phenotype-wide approach, pre-adult ADHD was associated with later occurrence of substance abuse, mood disorders, neurotic disorders, epilepsy, and poisoning. For those less well-examined health outcomes for which we had a working hypothesis – for example, bacterial/viral infection, road traffic accidents, and falls – while risk was significantly elevated in people with ADHD in basic analyses, there was marked attenuation to the point of non-significance after poverty and educational performance were added to the multivariable model.

### Comparison with existing studies

Our results add to the rapidly growing, if currently modest evidence base on the health consequences of ADHD. Consistent with other studies, we found a higher occurrence of mood disorders (depression) [15, 16], anxiety (neurotic) disorders [17], and substance abuse [18] in people with ADHD relative to their unaffected peers. We did not have a sufficiently high number of people with substance use disorders to disaggregate this endpoint further. Meta-analyses suggest that, relative to population controls, people with ADHD are three times as likely to be nicotine-dependent [32] and have a drug use disorder [33] which includes marijuana [34] and alcohol [15]. Several psychological processes could explain the higher burden of these outcomes in the ADHD-affected, including self-medication whereby nicotine enhances mental focus and ethanol alleviates feelings of restlessness, hyperactivity and anxiety [35].

We also found a raised risk of adult epilepsy in people diagnosed with ADHD in childhood/adolescence. Studies in this area have traditionally examined the role of epilepsy in the development of ADHD rather than the converse [36]. In a rare exception, reports from a Taiwanese cohort also generated from registry linkages, suggest ADHD was associated with a four-fold elevation in the risk of this neurological condition in later life [37]. While pathways connecting epilepsy and ADHD have yet to be identified, there may be overlapping pathophysiologic and genetic mechanisms at play. For instance, in animal experimentation, a small group of rats bred to be seizure-prone spontaneously developed behavioural characteristics redolent of those seen in people with ADHD [38].

Lastly, in our study, people with ADHD had a higher likelihood of experiencing poisonings requiring hospital attention. Ascribing intention to episodes of poisonings is challenging for an attending-physician, however, those of deliberate [39] and unknown aim [40] have both be shown to be more common in people with ADHD in meta-analyses and large-scale administrative datasets, respectively. Deliberate poisoning accords with the literature on the elevated burden of self-harm and attempted suicide in people who are neurodiverse, particularly those with ADHD [41]. When accidental, this could be a product of lower risk perception in people with an ADHD diagnosis who, on average, have lower cognitive function relative to the general population [23]. This explanation has been advanced for the elevated risk of unintentional injury in people who achieve lower scores on standard IQ tests [42]. In controlling for educational performance, a close correlated of IQ [43], the magnitude of the ADHD–poisoning relation, while clearly attenuated, was not eliminated. It may therefore simply be the core ADHD symptoms of hyperactivity, impulsivity, and, particularly, inattention that is driving a higher burden of unintentional injury in ADHD diagnosis.

### Study strengths and limitations

Our study has some strengths. These include the use of a full nation birth cohort with complete linkage to health records, so minimising concerns regarding selection bias and generalisability. Second, in our analyses we were able to replicate well-established findings in ADHD population-based research: a higher prevalence of being male, socioeconomically deprived, and diminished academic performance in individuals with a diagnosis of this neurodevelopmental condition. These characteristics are also known predictors of total mortality [44, 45] – again, observations made in the present dataset. Collectively, these findings give us confidence in the newer relationships reported here for ADHD and the occurrence of specific health conditions. Third, as illustrated by systematic reviews of ADHD and injury risk [46], the large majority of studies rely on self-reported information on ADHD and health outcomes, so raising concerns about data validity. By contrast, our analyses were based on physician-verified data on exposure and outcome.

Our work is of course not without its limitations. First, all diagnoses – ADHD and health outcomes – were based on conditions severe enough to warrant hospital attention, whether in- or out-patient; people with less severe ADHD symptoms were therefore not captured. That the period prevalence of ADHD herein is based on administrative data may lead to some under-detection, such that studies which include sample-wide assessment of ADHD symptoms yield higher estimates [10]. Second, relatedly, it is unclear if symptoms below current diagnostic thresholds for ADHD have an impact on adverse health events. Having data on self-reported symptoms captured via validated questionnaire [47] – while not without the described concerns of validity – alongside a derived polygenic risk score where DNA is available [48] would facilitate such dose-response analyses to test the hypothesis that a greater severity of symptoms is associated with a higher burden of a given health endpoint. Having data on symptoms of attention deficit separate to hyperactivity would also facilitate examination of the impact of each characteristic on health and injury. Third, although we only computed effect estimates for those health endpoints with ten or more events, the breadth of the resulting confidence intervals suggests some analyses were underpowered. Fourth, in an attempt to ascertain independence of association, we adjusted for educational performance of the study member; this is moot given that this characteristic may lie on mechanistic pathway connecting ADHD with injury and disease. Lastly, out-patient care episodes, including those for ADHD, were recorded from 1998 when study members were aged 11. As such, people with a prior ADHD diagnosis who did not continue to seek hospital care would not have been included in the ADHD group.

### Future research directions

ADHD, in contrast to other neurodevelopmental disorders such as autism and intellectual disability, is eminently treatable pharmacologically with psychostimulants, amongst other medications, and also psychosocial intervention which includes cognitive behavioural therapy [49]. Well-controlled observational studies and clinical trials reveal that when core symptoms are successfully treated there is a concomitant positive impact on educational performance [50] and quality of life [51], whereas discontinuation of therapy leads to a decline in grade point average [52]. Whether treatment effects extend to the range of health outcomes associated with ADHD herein – as apparent for suicide attempt [53], unintentional injury [22, 54], and criminality [55] – requires further study and we did not have data on prescriptions dispensed to investigate the impact of education.

While correlations between a growing array of adverse health outcomes and antecedent ADHD have been reported, in these cohort studies, like our own, data are generated using linked  electronic health records. Such studies are typically not well-equipped to examine the mechanisms underpinning these associations. For this to occur, field-based cohorts with higher resolution measurement of risk indices such as health behaviours (e.g., alcohol intake, physical activity) and biomarkers (e.g., inflammation, blood glucose) are required.

In conclusion, children and adolescents with ADHD in the present study had an increased future burden of psychological and neurological conditions but not somatic disorders.

## Supplementary information

Supplemental file
