# Supplementary material for: Pre-adult attention-deficit/hyperactive disorder and later adverse health outcomes: the 1987 Finnish birth cohort study
Source: Mol Psychiatry. 2026 Jul 13;31(11):6310–6. doi: 10.1038/s41380-026-03700-6 (PMC13668644; doi:10.1038/s41380-026-03700-6)
Supplement: Supplementary file 1 [file 41380_2026_3700_MOESM1_ESM.docx]

**Supplemental file**

**Batty et al. Molecular Psychiatry: "Pre-adult attention-deficit/hyperactive disorder and later adverse health outcomes: The 1987 Finnish birth cohort study"**

Supplemental Table 1. Comparison of statistical significance for the association of ADHD and health outcomes using the Bonferroni and Benjamini-Hochberg corrections

Supplemental Table 2. Study member characteristics in relation to total mortality in the 1987 Finnish Birth Cohort Study (N=59476)

Supplemental Table 3. Study member characteristics according to inclusion in the analytical sample: The 1987 Finnish Birth Cohort

Supplemental Figure 1A. Odds ratios (99.6% confidence interval) for the association between

ADHD diagnosis and subsequent hospitalisation for 12 health conditions – follow-up between age 18 and 25.5 years

Supplemental Figure 1B. Odds ratios (99.5% confidence interval) for the association between ADHD diagnosis and subsequent hospitalisation for 11 health conditions – follow-up between age 25.5 and 33 years

Supplemental Figure 2A. Odds ratios (99.5% confidence interval) for the association between

ADHD diagnosis and subsequent hospitalisation for 11 health conditions – follow-up between age 18 and 25.5 years

Supplemental Figure 2B. Odds ratios (99.5% confidence interval) for the association between

ADHD diagnosis and subsequent hospitalisation for 10 health conditions – follow-up between age 25.5 and 33 years

Supplemental Figure 3A. Odds ratios (99.7% confidence interval) for the association between

ADHD diagnosis and subsequent hospitalisation for 14 health conditions using the imputed sample (N=54045)

Supplemental Figure 3B. Odds ratios (99.7% confidence interval) for the association between

ADHD diagnosis and subsequent hospitalisation for 17 health conditions using the imputed sample (N=54045)

**Supplemental Table 1. Comparison of statistical significance for the association of 17 ADHD and health outcomes using the Bonferroni and Benjamini-Hochberg corrections**

|  | **Odds ratio**  **(99.7% confidence interval)** | **Benjamini-Hochberg**  **p-value** | **Statistical significance** | |
| --- | --- | --- | --- | --- |
|  |  |  | **Bonferroni** | **Benjamini-Hochberg** |
| F30-F39 Mood disorders | 2.45 [1.78;3.33] | 0.000 | Yes | Yes |
| G40-G42 Epilepsy | 4.55 [2.55;7.53] | 0.000 | Yes | Yes |
| F40-F48 Neurotic disorders | 2.11 [1.50;2.90] | 0.000 | Yes | Yes |
| F10-F19 Disorders due to substance abuse | 2.26 [1.56;3.19] | 0.000 | Yes | Yes |
| T36-T65 Poisoning | 2.30 [1.38;3.64] | 0.002 | Yes | Yes |
| M60–M79 Soft tissue disorders | 1.71 [1.16;2.45] | 0.014 | No | Yes |
| F20-F29 Psychotic disorders | 2.08 [1.17;3.42] | 0.017 | No | Yes |
| S00-T35 Injury | 1.43 [1.10;1.88] | 0.017 | No | Yes |
| A01-A79 Bacterial infections | 1.59 [1.08;2.28] | 0.028 | No | Yes |
| A80-B34 Viral infections | 1.84 [1.06;2.98] | 0.034 | No | Yes |
| M50-M51 Sciatica | 1.84 [0.99;3.12] | 0.053 | No | No |
| V01-V99 Road accidents | 1.56 [1.00;2.32] | 0.056 | No | No |
| W00-W19 Falls | 1.35 [0.97;1.84] | 0.089 | No | No |
| L00-L08, L20-L30 Infections and eczema | 1.22 [0.78;1.84] | 0.429 | No | No |
| G43-G44 Headaches | 1.25 [0.64;2.21] | 0.534 | No | No |
| J09-J18 Influenza and Pneumonia | 1.23 [0.63;2.15] | 0.547 | No | No |
| M54 Back pain | 1.05 [0.65;1.62] | 0.820 | No | No |

**Supplemental Table 2. Study member characteristics in relation to**

**total mortality in the 1987 Finnish Birth Cohort Study (N=59476)**

|  | **Total mortality** | |
| --- | --- | --- |
|  | **N events / N at risk** | **OR (95% CI)** |
|  |  |  |
| Male | 459 / 30435 | 2.95 [2.46-3.56] |
| Female | 150 / 29041 | 1.0 (ref) |
|  |  |  |
| Low grade point average | 294 / 14821 | 2.37 [2.01-2.79] |
| High grade point average | 315 / 44655 | 1.0 (ref) |
|  |  |  |
| Months claiming social income (per SD higher) | 609 / 59476 | 1.28 [1.21-1.34] |
|  |  |  |
| ADHD diagnosis | 8 / 279 | 2.15 [0.97-4.08] |
| ADHD free | 601 / 59197 | 1.0 (ref) |
|  | | |

ORs are sex-adjusted except in the analyses of risk according to

sex where they are unadjusted. For GPA, ORs are for low (4-7)

versus high [7-10]. Follow-up was to 2020. A SD increase in duration

claiming social income is 45.7 months.

**Supplemental Figure 1A. Odds ratios (99.6% confidence interval) for the association between**

**ADHD diagnosis and subsequent hospitalisation for 12 health conditions – follow-up between age 18 and 25.5 years**

Odds ratios are the odds of a given outcome in people with an ADHD diagnosis versus the those without (the referent).

Model 1 comprises sex; Model 2 sex, family socioeconomic status, and birth weight; and Model 3 all described variables

plus educational performance.

As a birth cohort, there is no variation in age in the study sample, so no such adjustment was required.

All effect estimates are corrected for multiple testing.

Percent attenuation is a comparison of effect estimates in Model 3 and Model 1.

**Supplemental Figure 1B. Odds ratios (99.5% confidence interval) for the association between ADHD diagnosis**

**and subsequent hospitalisation for 11 health conditions – follow-up between age 25.5 and 33 years**

Odds ratios are the odds of a given outcome in people with an ADHD diagnosis versus the those without (the referent).

Model 1 comprises sex; Model 2 sex, family socioeconomic status, and birth weight; and Model 3 all described variables

plus educational performance.

As a birth cohort, there is no variation in age in the study sample, so no such adjustment was required.

All effect estimates are corrected for multiple testing.

Percent attenuation is a comparison of effect estimates in Model 3 and Model 1.

**Supplemental Figure 2A. Odds ratios (99.5% confidence interval) for the association between**

**ADHD diagnosis and subsequent hospitalisation for 11 health conditions – follow-up between age 18 and 25.5 years**

Odds ratios are the odds of a given outcome in people with an ADHD diagnosis versus the those without (the referent).

Model 1 comprises sex; Model 2 sex, family socioeconomic status, and birth weight; and Model 3 all described variables

plus educational performance.

As a birth cohort, there is no variation in age in the study sample, so no such adjustment was required.

All effect estimates are corrected for multiple testing.

Percent attenuation is a comparison of effect estimates in Model 3 and Model 1.

**Supplemental Figure 2B. Odds ratios (99.5% confidence interval) for the association between**

**ADHD diagnosis and subsequent hospitalisation for 10 health conditions –follow-up between age 25.5 and 33 years**

Odds ratios are the odds of a given outcome in people with an ADHD diagnosis versus the those without (the referent).

Model 1 comprises sex; Model 2 sex, family socioeconomic status, and birth weight; and Model 3 all described variables

plus educational performance.

As a birth cohort, there is no variation in age in the study sample, so no such adjustment was required.

All effect estimates are corrected for multiple testing.

Percent attenuation is a comparison of effect estimates in Model 3 and Model 1.

**Supplemental Figure 3A. Odds ratios (99.7% confidence interval) for the association between**

**ADHD diagnosis and subsequent hospitalisation for 14 health conditions using the imputed sample (N=54045)**

Odds ratios are the odds of a given outcome in people with an ADHD diagnosis versus the those without (the referent).

Model 1 comprises sex; Model 2 sex, family socioeconomic status, birth weight and parental cohabitation;

and Model 3 all described variables plus educational performance.

As a birth cohort, there is no variation in age in the study sample, so no such adjustment was required.

All effect estimates are corrected for multiple testing.

Percent attenuation is a comparison of effect estimates in Model 3 and Model 1.

**Supplemental Figure 3B. Odds ratios (99.7% confidence interval) for the association between**

**ADHD diagnosis and subsequent hospitalisation for 17 health conditions using the imputed sample (N=54045)**

Odds ratios are the odds of a given outcome in people with an ADHD diagnosis versus the those without (the referent).

Model 1 comprises sex; Model 2 sex, family socioeconomic status, birth weight and parental cohabitation;

and Model 3 all described variables plus educational performance.

As a birth cohort, there is no variation in age in the study sample, so no such adjustment was required.

All effect estimates are corrected for multiple testing.

Percent attenuation is a comparison of effect estimates in Model 3 and Model 1.
